# Ultrasonic intensification as a tool for enhanced microbial biofuel yields

**DOI:** 10.1186/s13068-015-0321-0

**Published:** 2015-09-15

**Authors:** Balakrishnan Naveena, Patricia Armshaw, J. Tony Pembroke

**Affiliations:** Molecular Biochemistry Laboratory, Materials and Surface Science Institute, Department of Chemical and Environmental Sciences, University of Limerick, Limerick, Ireland

**Keywords:** Algal and cyanobacterial biofuels, Ultrasonic intensification, Yield enhancement, Ultrasonic reactors

## Abstract

Ultrasonication has recently received attention as a novel bioprocessing tool for process intensification in many areas of downstream processing. Ultrasonic intensification (periodic ultrasonic treatment during the fermentation process) can result in a more effective homogenization of biomass and faster energy and mass transfer to biomass over short time periods which can result in enhanced microbial growth. Ultrasonic intensification can allow the rapid selective extraction of specific biomass components and can enhance product yields which can be of economic benefit. This review focuses on the role of ultrasonication in the extraction and yield enhancement of compounds from various microbial sources, specifically algal and cyanobacterial biomass with a focus on the production of biofuels. The operating principles associated with the process of ultrasonication and the influence of various operating conditions including ultrasonic frequency, power intensity, ultrasonic duration, reactor designs and kinetics applied for ultrasonic intensification are also described.

## Background

The utilisation of algae and cyanobacteria as cell factories for the production of biofuels has recently received considerable attention. Their ability to grow photoautotrophically and provide a renewable and sustainable biofuel source whilst also providing additional opportunities for co-product biorefinery makes them intriguing candidates [1]. There have been many reports that algal biofuel production is technically if as yet not economically viable [2–5]. Therefore, any technique that improves the efficiency of biofuel production is likely to have a significant effect on the viability of these production processes.

Algae and cyanobacteria can be metabolically engineered to produce biofuels such as ethanol [6, 7], biogas [8], hydrogen [9, 10], free fatty acids for transesterification to biodiesel [11, 12] and butanol [13]. Furthermore, there are several ways to convert algal or cyanobacterial biomass to fuel energy such as biochemical conversion, chemical treatment, direct combustion and thermochemical conversion [14]. Lipids can be extracted and transesterified into biodiesel while starch, glycogen and carbohydrate polymers, can be further hydrolyzed and converted into bio-ethanol [14]. Indeed following lipid extraction from *Chlorococum sp*. the residual biomass, used as a feedstock, has been shown to produce significant amounts of bioethanol upon fermentation with concentrations of 3.83 g L^−1^ achievable [15, 16]. In this case, integration of an ultrasonication pre-treatment, known to enhance lipid extraction yields and biogas production can also be quite beneficial in disrupting the spent biomass [8, 17, 18]. This review aims to examine the beneficial aspects of ultrasonic intensification of lipid extraction, fermentation and production of biofuels.

### Ultrasonic process intensification: concept introduction

**Fig. 1:**
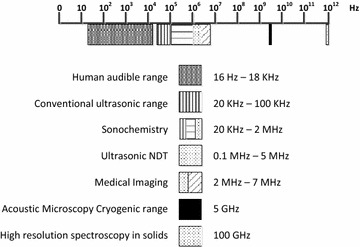
Ultrasonic frequency scale for various fields of technology

Ultrasonication is a branch of acoustics that can be applied to solids, liquids and gases at frequencies above the human hearing range [19]. The frequencies applied for processes are shown in Fig. 1. Particle agitation in a liquid culture can be achieved by applying acoustic energy with frequencies ranging from 10 kHz to 20 MHz using ultrasonic probes, an ultrasonic bath, a flat plate or a tube type ultrasonicator [20]. The process operates by converting electrical energy into physical vibration which directly influences the medium it is applied to by imparting high energy to the medium via cavitation [21]. During the vibration process, the microbubbles present in the form of nuclei are increased in size to a maximum of about 4–300 mm in diameter [22], and can be either stable or transient. In the case of low ultrasonic intensity, the radii of microbubbles periodically and repetitively expand and shrink causing radial oscillation within several acoustic cycles. At the point when acoustic energy has sufficient intensity, some microbubbles become unstable and when the resonant frequency of bubbles exceeds that of the ultrasonic field, the bubbles collapse within a few nanoseconds at the solid/solvent interface (>200 mm) [21] which produces microjets with a velocity >100 m s^−1^ and shock waves of approximately 103 MPa towards the solid surface of the substance in solution. This causes cavitation of the substance in the liquid medium, with the violent movement of fluid towards or away from the cavitational microbubbles defined as micro-convection [21]. The convection in the ultrasonic system has two components, i.e., microturbulence or micro-convection and shock waves [23]. The authors have stated that micro-convection is a continuous oscillatory motion of liquid medium induced by radial movement of cavitation bubble and governs the growth of the nuclei while shock waves are discrete, high pressure amplitude waves emitted by the bubble which increase the nucleation rate [23]. This micro cavitation influences the transport of fluids and solid particles within the medium and results in forces that can cause emulsification or dispersion, while the strong shockwaves and microjets generate extremely strong shear forces over those of conventional mechanical methods, and are able to scatter liquid into tiny droplets or crush solid particles into fine powders [21].

Ultrasonication which enhances biochemical reactions is termed ultrasonic process intensification [22] and can be differentiated into low and high intensity applications. In several biotechnology processes, both high and low intensity ultrasonic waves have been utilised depending on the objective of the sonication process [24]. Low intensity ultrasonic (<1 W cm^−2^ and between 1 and 10 MHz) intensification can be considered non-destructive as it sends ultrasonic waves through a liquid medium without causing any permanent physical or chemical change in the medium or microorganisms within. The low intensity ultrasonication can also be defined in terms of acoustic pressure amplitude. When the acoustic pressure amplitude is less than the static pressure in the medium, the bubbles undergo stable, small amplitude radial motion called “stable cavitation”. The bubble motion turns transient when the acoustic pressure amplitude exceeds the static pressure in the medium. The microorganisms in the medium respond to the low energy only during the time of exposure to the ultrasonic waves and return to an equilibrium state when the ultrasonic source is removed [24]. On the other hand, high intensity ultrasonic intensification or low frequency ultrasonication (10–1000 W cm^−2^ and 10–100 kHz) which generates high pressure in the medium can disrupt microbial cellular structures [24]. This can cause the lysis of microbial cells or the formation of free radicals in chemical degradation reactions [25]. Thus, low intensity ultrasonic intensification is quite distinct and transitory when applied periodically. The type of cavitation generated during ultrasonic process intensification depends on several parameters, including amplitude, pressure, temperature, viscosity and concentration of the medium [26].

## Physical mechanism of ultrasound assisted processes for biofuels production

Ultrasound causes both physical and chemical changes through the process of cavitation. Chemically, highly reactive radicals can be generated from the dissociation of the entrapped vapour molecules in a cavitation bubble [27]. Physically, cavitation can cause intense convection in a bulk medium leading to microturbulence (an intense oscillating motion of liquid with low to moderate velocities) and shock waves (high pressurised waves emitted by the bubble, with amplitudes as high as 30–50 bar). During lipid extraction from biomass, the physical effects of ultrasonication can significantly enhance the lipid yield. Microturbulence can lead to a more efficient mixing of the biomass and solvent (without induction of shear stress), while shock waves can cause rupture of the cell wall [28]. Ultrasound can also generate intense local turbulence in the medium, pushing the extracted lipids away from the surface of the microbial cells, and thus, maintaining a constant concentration gradient for continuous diffusion of lipids from the cells [27].

The physical effects of ultrasonication can also enhance the transesterification process during biodiesel production [29–32]. Ultrasonication generates an enormous interfacial area between the oil and alcohol due to microturbulence leading to the formation of fine emulsions [33]. Kalva et al. [29] investigated the mechanism of the enhancement of transesterification by discriminating the physical and chemical effects of ultrasound. This was analysed by transesterifying soybean oil using methanol and sodium hydroxide as the base catalyst with ultrasound frequency set at 20 kHz and power output of 100 W. It was reported that the enhancement of transesterification was due to the physical effect rather than a chemical effect of ultrasound, i.e., production of radical species and acceleration of the reaction by these species [34–36]. In addition it was found that the transesterification yield increased when the alcohol to oil molar ratio was increased. This was due to the effect of low intensity microturbulence generated by the cavitation bubbles in the oil, which restricted the dispersion of oil in methanol at high alcohol to oil molar ratios [29].

Ultrasound may also enhance enzymatic reactions within the cell. Khanna et al. [30] reported that the nature of convection generated by ultrasound at raised static or ambient pressure in the medium provided oscillatory motion of liquid, accompanied by medium amplitude movement by the acoustic or shock waves emitted by the bubbles. This was concluded by investigating the mechanism of enhancement of glycerol conversion into 1,3-propanediol and ethanol using *Clostridium pasteurianum* MTCC 116 with the aid of ultrasonication. Mild shock waves of ultrasonication were shown to cause rapid movement of microbial cells in the fermentation broth. Inter-collisions and/or collisions with the walls of the test tube lead to the acceleration of enzymatic reactions inside the cells [30]. In addition, it was reported that the high velocity micro-streaming caused desorption of the CO_2_ produced in the metabolic pathway and thus favoured a faster diffusion of glycerol into the microbial cells. As a result, the overall enzymatic reaction system is expected to be substrate saturated with enzyme being the limiting reactant. During ethanol formation in this system the Michaelis constant (K_m_) was found to decrease with ultrasonication indicating a rise in the formation of enzyme–substrate complex. Hence, ethanol yields were found to increase from 0.024 to 0.04 mol mol^−1^ (i.e., 83 % increase) and a decrease in substrate inhibition (K_I_) contributed to the enhancement of 1,3-propanediol yields from 0.017 to 0.021 mol mol^−1^ (30.89 % increase) [30, 36].

Ultrasonication has also been shown to enhance ethanol fermentation processes by increasing transport through the cell membrane and increasing enzymatic hydrolysis with strong micro-convection [37–40]. Recent studies have found that it could enhance the utilisation of substrate for cell growth, reduce the inhibition by substrate and decrease the specific death rate of cells leading to an increase of ethanol yield from 0.149 to 0.166 g g^−1^ of raw biomass [41].

## Ultrasonic intensification applicability within extraction processes

Traditionally, solvent based processes are utilised for extraction of many bioactive compounds [42, 43]. However, standard extraction mechanisms such as cell maceration and soxhlet extraction have limitations such as high solvent consumption, large operating cost and extended operation times which frequently result in lower yields [44]. The application of ultrasonic intensification can allow higher yields to be generated in a shorter time, with lower energy input [45] and without adding additional reagents to the extraction. In addition, the effects of increasing temperature on the extraction components can be avoided. Such modified extraction techniques have been developed recently for the extraction of macromolecules such as polysaccharides, proteins, terpenoids, flavonoids, carotenoids and phenolic compounds [46–49]. The ultrasonic intensification process can be effectively used to improve the extraction rate by increasing the mass transfer due to the formation of microcavities leading to higher growth and product yields. Ying et al. [49] reported that ultrasonic-based extraction is associated with two main physical phenomena, acoustic cavitation and diffusion through the cell wall. The conditions associated with cavitation, an increase in temperature and pressures up to 100 MPa, produce very high shear energy waves and turbulence in the cavitation zone. The combination of these factors (pressure, heat and turbulence) is used to accelerate mass transfer in the extraction process. Ultrasonic intensification also exerts a mechanical effect which leads to enhanced diffusion of solvents into the cell wall. In pure liquids, the microbubbles retain their spherical shape during the collapse, as their surroundings are uniform [50]. However, when the microbubbles collapse near a solid surface it occurs asymmetrically and produces shock waves toward the cell wall. These waves have a strong impact on the cell surface; therefore, enhance the solvent penetration into the cell. Another effect caused by the ultrasound wave is that it can facilitate the swelling and hydration of biomass and so cause an enlargement of pores in the cell wall which can improve diffusion processes and therefore enhance mass transfer which can enhance extraction yield [50]. Hence ultrasonic intensification can provide high extraction efficiency in a short time with less solvent consumption over other extraction techniques [51]. As an example, Rocco et al. [52] reported a 73 % increase in recovery of polychlorinated biphenyls from biomass generated through treatment of household wastewater with 30 min of ultrasonic intensification during the extraction process.

## Benefits of ultrasonic intensification for lipids extraction in biofuel production systems

Algae and cyanobacteria are of interest as a renewable energy source for biofuel largely because of their autotrophic lifestyle and their potential to produce a range of additional products which can aid the economics of biofuel production [53]. For lipid production in particular, lipid extraction is required and cell disruption is necessary for lipid recovery [54]. Though several disruption methods including compression, high pressure homogenization, autoclaving, bead mill treatment, microwave treatment and magnetic stirring have been employed, both at laboratory and pilot scale, the energy requirement for those techniques is typically higher than the energy of combustion of lipid extracted [55]. See Table 1 for a comparison of these extraction methodologies.

**Table 1:** Comparison of various algal lipid extraction methods

Strain	Method of lipid extraction	Research findings	Remarks	References
*Botryococcus braunii*	Switchable hydrophilicity solvent, *N*,*N*-dimethylcyclohexylamine	The solvent extracted up to 22 wt% of crude lipid from the algal biomass	Drawbacks include toxicity, poor selectivity, contaminants separation	[56]
*Scenedesmus obliquus* *Chlorella protothecoides* *Nannochloropsis salina*	Supercritical fluid method using CO_2_	The maximum extraction yield 18.15 wt% was obtained at 60 °C and 30 MPa with 0.4 kg.h^−1^ of CO_2_ and 5 % of co-solvent (ethanol). *Scenedesmus obliquus* oil was found to be rich in ω-3 fatty acids	Environmental and safety issues in large scale extraction.	[57]
*Botryococcus* sp. *Chlorella vulgaris* *Scenedesmus sp*.	Autoclaving at 125 °C with 1.5 MPa for 5 min	The lipid content of the three species was in the range of 5.4–11.9 wt%	Poor extraction efficiency	[58]
*Botryococcus* sp. *Chlorella vulgaris* *Scenedesmus* sp.	Bead-beating	The lipid content of three species was in the range of 7.9–8.1 wt%	Difficult to scale up	[58]
*Botryococcus* sp. *Chlorella vulgaris* *Scenedesmus* sp.	Microwave	The lipid content of three species was in the range of 10.0–28.6 wt%	Easy scale-up high energy demand for cooling maintenance cost at scale.	[58]
*Botryococcus* sp. *Chlorella vulgaris* *Scenedesmus* sp.	Osmotic shock by 10 % NaCl solution	The lipid content of three species was in the range of 6.8–10.9 wt%	Requires longer treatment time.	[58]
Mixed algal culture of *Scenedesmus* sp., *Chlorococcum* sp.	Sonication	The ultrasound application enhanced the lipid extraction yield by about 96 % (26.8 wt% lipids extracted from 57.6 % of total biomass)	Reduced extraction time and increased lipid extraction efficiency	[59]

Ultrasonic intensification has been shown to improve lipid extraction via cell disruption with more favourable economics than other disruption methods [60], enhancing the extraction of lipids by 50–500 % compared to traditional methods, with a 10 fold reduction in extraction time [61]. Suganya and Renganathan [62] investigated lipid extraction from the green algae *Ulva lactuca* using ultrasonication and found that the yield of lipid (8.49 %), was maximised with a 6 min ultrasonic pre-treatment. Lipid extraction with *Synechocystis aquatilis* also demonstrated that ultrasonication resulted in higher yields of lipid, with 21.30 % extracted compared to grinding (18.74 %), osmotic shock (14.55 %) and non-disruptive methods (10.17 %) [63]. For more examples, see Table 2 detailing the reaction conditions and lipid yields recovered from various microalgae sources using ultrasonic intensification.

**Table 2:** Reaction conditions for ultrasonic intensification on extraction of lipids from various microalgae

Microalgae	Reaction parameters				Yield (%) (v/w)	References
	Frequency (kHz)	Solvent	Methodology	Time (min)		
*Chlorella vulgaris*	10	CHCl_3_-MeOH (1:1) (v/v)	Finely ground dried biomass with distilled water was subjected to microwave oven (Sharp R-15AT 1000 W; 2450 MHz: 150 s) and 5 min ultrasonication. The lipids were extracted with the solvent: biomass ratio of 2:1. The mixture was again ultrasonicated for 30 min	30	9.82	[64]
*C. minutissima*	10	*n*-Hexane	Dried biomass (100 mg) with 20 mL of *n*-hexane was subjected to ultrasonic bath and further centrifugated (4500 rpm for 10 min). Supernatant and cell debris were removed separately.	20	15.5	[60]
*Thalassiosira fluviatilis*	10	*n*-Hexane		20	40.3	[60]
*T*. *pseudonana*	10	*n*-Hexane		20	39.5	[60]
*Chlorella* sp.	20	Solvent free	700 mL of wet culture was ultrasonicated directly without dewatering using an ultrasonic system [ultrasonic processor, sonotrode, water cooling jacket for the flow cell (100 mL), recirculation tank for feed and processed sample storage and a centrifugal pump for sample circulation]	Continuous system	75	[65]

Algal cell walls are typically tri-layered rigid structures with high tensile strength [66], hence the release of intra lipids can be blocked. Homogenization can affect the outer cell walls with shearing force but not the interior of the cell. Extraction of lipids from cells may occur by either diffusion of lipids across the cell wall, if the algal biomass is suspended in the solvent with higher selectivity and solubility (or large partition coefficient) for lipids or disruption of the cell wall with release of cell contents in the solvent. The diffusive mechanism is less efficient due to slow diffusion of lipid across the cell wall while disruptive mechanism results in faster extraction with high yield as it causes direct release of lipid due to the rupture of cell wall [27]. Sonication can interfere with the cell interior via shock waves produced by imploding cavitation bubbles [67]. Thus, ultrasonic intensification can be an advantageous addition for lipid extraction processes involving algae. Park et al. [68] investigated the effect of homogenisation and ultrasonication in combination on lipid extraction from *Chlorellavulgaris*. The initial fatty acid content of *C*. *vulgaris* was 360 mg g^−1^ cell. Lipid recovery was found to increase when both techniques were combined compared with the use of either alone. The results of the combined processes showed 100.5, 123.9, and 152.0 mg lipid g^−1^ cell recovered for the 20, 40, and 60 min reaction times used, respectively. The yields were 5.3-fold, 6.6-fold, and 8.1-fold higher, respectively, than that of the control (single treatment by sonication or single treatment via homogenization). In this system [68], microalgal suspensions were allowed to circulate continuously between the homogenizer and ultrasonicator and it was concluded that cell walls damaged slightly by homogenization would be effectively disrupted by the subsequent ultrasonication-induced cavitation bubbles resulting in a superior extraction process [68]. When the cell concentration was increased to 40 g.L^−1^, the lipid recovery yield for 1 h of sonication-assisted homogenization was increased to a very high 281.3 mg lipid g^−1^ cell using the chloroform–methanol solvent. This system’s treatment of high cell concentrations makes it possible to enhance lipid recovery capacity based on unit time [68].

In addition to enhancing the yield of lipids extracted from cells, low frequency ultrasonic intensification can also enhance emulsion generation with immiscible liquids which are commonly used during biodiesel production. This was confirmed via an alkali catalysed biodiesel production process performed by Ji et al. [69] which found that a 100 % emulsion could be achieved within 20 min with the utilisation of a jacketed ultrasonic reactor [69]. Moreover, the beneficial effects of ultrasonication have been reported for the transesterification of triglycerides with methanol. Reaction times could be reduced, as could the catalyst requirement, the energy consumption and the alcohol to oil molar ratio [70, 71]. Because of these beneficial effects on extraction and transesterification, these processes are frequently carried out concurrently. When both lipid extraction and transesterification are carried out simultaneously, it is referred to as in situ transesterification [72]. In situ transesterification processes using microbial biomass for biodiesel production can greatly enhance the biodiesel yield, as described in Table 3.

**Table 3:** Effect of ultrasonic intensification on in situ transesterification of various microbial biomasses

Source	Catalyst	Process parameters				Biodiesel yield (%)	References
		Solvent: biomass ratio (v/w)	Frequency (kHz)	Temperature (°C)	Time		
*Chlorella vulgaris*	KF/CaO	8:1	40	60	45 min	63.49	[73]
*Enteromorpha compressa* (macroalgae)	H_2_SO_4_	5.5:1	40	70	90 min	98.89	[74]
*Chlorella* sp.	H_2_SO_4_	79:1	24	60	8 h	99.9	[75]
*Trichosporon oleaginosus* (Yeast)	NaOH	60:1	20	25	12 h	92.1	[72]
*Scenedesmus* sp.	Tungstated zirconia (WO_3_/ZrO_2_)	60:1	22.5	50	20 min	71.37	[76]

## Ultrasonic intensification, utility in fermentation processes

High and low intensity ultrasonic intensification methods are utilised to enhance industrial fermentation processes including those involving the production of biofuels [73]. Ultrasonic intensification has been used as a pre-treatment prior to the fermentation of algal biomass for the production of bioethanol [74]. Upon ultrasonication, significant changes in the physicochemical properties of the algal cell enhance the bio-accessibility of carbohydrate substrates in the fermentation media, increasing their availability for microbial fermentation. Sonication pre-treatments (frequency 40 kHz; power output 2.2 kW) for 15 min or longer on *Scenedesmus obliquus* YSW15 resulted in increasing the concentration of dissolved carbohydrate. This increased availability resulted in enhanced ethanol production by up to 5.6 g L^−1^. In addition, sonication can decrease algal surface hydrophobicity and increase the electrostatic repulsion among the algal debris dispersed in the aqueous solution. This can provide more facile access to the treated algal biomass, enhancing the assimilation of algal carbohydrates by anaerobic bacteria isolated from seed sludge of municipal wastewater [74]. Low intensity ultrasonication has also been found to promote protein production and increase cell concentration through the enhancement of cell membrane porosity [75]. For instance, upon ultrasonication, *Pseudomonas aeruginosa* membrane porosity has been shown to increase, resulting in the enhanced uptake of 16-doxylstearic acid (a hydrophobic antibiotic) through the cell membrane [76]. In general, such increases in cell permeability lead to enhanced diffusion rates and thereby increase the overall cell productivity and growth rate [77]. In the case of *E*. *coli*, the average cell count was almost doubled by the inclusion of an ultrasonication treatment [8.5 × 10^7^ CFU ml^−1^ versus 4.8 × 10^7^ CFU ml^−1^] [77]. Indeed the lag time (λ) for non-ultrasonicated biomass during H_2_ production from the microalgae *Scenedesmus obliquus* YSW15 was higher than that for the ultrasonicated biomass. λ derived from the Gompertz equation indicated that the non-ultrasonicated biomass had an average 9 h lag phase, which was reduced to 6 h after ultrasonication (45 kHz with power output 2.2 kW). The shorter lag phase in ultrasonicated biomass fermentation was reported due to the easy accessibility of dissolved carbohydrate to fermentative bacteria compared with the non-sonicated control [78]. The maximum H_2_ production was obtained from 60 min ultrasonicated biomass (2419 mL L^−1^) whereas the non-sonicated control and short-term ultrasonicated biomass for 5 min produced 1393 and 1370 mL L^−1^, respectively [78]. Ultrasound (at 20 kHz) has also been demonstrated to induce protein production, specifically thrombinase (a fibrinolytic enzyme) production from marine actinomycetes, likely due to increased mass transfer rates leading to increased nutrient uptake [79]. In conventional bioreactors, microbial cells suspended in the fermentation broth are invariably surrounded by a stagnant film of liquid [80]. This film can hinder the mass transfer of nutrients and products and can be a rate controlling factor [80]. In an ultrasonicated bioreactor, the pulsation of microbubbles of gas in the fluid generates micro-streaming [81] which can minimise the fluid boundary layer around cells located close to the bubbles [82], thus enhancing mass transfer. Ultrasonic intensification can also increase the rates of gas–liquid oxygen transfer, removal of carbon dioxide and dissolution of suspended solids. This can increase the supply of low solubility substrates and, indirectly, enhance microbial cell productivity [83]. Consequently, there are many benefits to the inclusion of a sonication treatment within fermentation processes. Ultrasonic contribution to the enhancement of fermentation yield is given in Table 4.

**Table 4:** Effect of ultrasonic intensification on fermentative bioenergy

Biomass	Inoculum	Ultrasonic extraction conditions			Ethanol/hydrogen yield	References
		Frequency (kHz)	Power (W)	Temperature (°C)		
*Parthenium hysterophorus*	*Saccharomyces cerevisiae* MTCC 170	35	35	30 ± 2	Ethanol: 0.85 g L^−1^ h^−1^	[37]
Waste paper	Klebsiella oxytoca	36	150	–	Ethanol: 0.38 g L^−1^ h^−1^	[84]
Oil palm fronds	*Saccharomyces cerevisiae*	37	20	75	Ethanol: 3.64 g L^−1^ h^−1^	[85]
Corn meal	*Saccharomyces cerevisiae var. Ellipsoideus*	40	–	60	Ethanol: 3.02 g L^−1^ h^−1^	[86]
*Chlorella vulgaris*	Anaerobic digested sludge	20	150	78	Hydrogen: 31.9–37.9 mL g^−1^ dry cell weight	[87]
*Chlamydomonas reinhardtii*	*Thermotoga neapolitana*	–	130	–	860 mL mL^−1^ culture	[88]

The length of ultrasonic treatment has a significant effect on product yields. In a study using *c* with both continuous and intermittent ultrasonication during fermentation it was found that the ethanol concentration was increased up to 30 g L^−1^ with intermittent ultrasonic intensification, whereas with continuous ultrasound intensification, no increase was observed [89]. Similarly intermittent ultrasonic intensification (in fermentation broth was found to enhance the production of a fibrinolytic enzyme from *Bacillus sphaericus* MTCC 3672 by 1.82 fold compared to non-sonicated broth [90]. The intensity of ultrasonication can also affect yields obtained. In a study with molasses fermentation by *Saccharomyces cerevisiae* M30, the effect of ultrasonic intensification on ethanol production was examined. The ultrasonic frequencies were varied as 20, 25 and 30 kHz with a maximum specific ethanol production rate of 1.55 g g^−1^ h^−1^ being achieved at 25 kHz [91].

While low to high intensity pulsing can enhance productivities, increased microbial exposure to ultrasonic power causes cells to become fragile and reduces microbial viability due to mechanical stress on the microbial cell [92]. The mechanical shear caused by the ultrasonic waves can disrupt the cell wall at higher duty cycles and ultimately has a lethal effect [93]. Thus, numerous studies have shown that intermittent ultrasonic intensification is more advantageous, increasing product yield while keeping microbial viability high. In addition, the life span of the ultrasonic transducers is increased and temperature effects due to the transducer usage are minimised [94]. Thus, examination of the effects and the rates of ultrasound intensification utilised appear to be important for individual process strategies.

## Reactor design for ultrasonic intensification

The effect of intensification on a bioprocess depends on the choice of ultrasonic parameters utilised. These include the ultrasonic mode, which can be either continuous or pulse, the frequency, the intensity, the processing temperature, the nature of the solvent utilised, the aeration and the design of reactor which determines the level and distribution of energy within the system [19]. The main parameters in ultrasonic reactor design are the type of reactor, its geometry; the design of the transducer set up and the volumetric scale of the feed stock [19]. In addition, the location of the ultrasonic probe in the reacting vessel has been found to influence the distribution of cavitational behaviour within the system [95]. The probe location is usually dependent on the reactor size and the working volume of the liquid reactants [96]. The vertical location of probes in the reactor has been shown to result in poor distribution of cavitation microbubbles with only 10 % distribution reported in a 2.5 L reactor [97]. In contrast, the horizontal location of a probe in a reactor with 82 % immersion length was shown to result in uniform cavitational behaviour over the entire reactor [98]. Another important factor is the shape and diameter of the probe tip. Ultrasonic probes with large diameter tips provide low ultrasonic energy density owing to a wide emitter surface [99]. Usage of larger tips allows higher reaction rates to be achieved. In a study utilising *B. amyloliquefaciens* producing α-amylase, rapid deactivation of α-amylase was observed to occur, due to the reaction between hydroxyl radicals and the enzyme but this was shown not to take place inside the bubbles. High density cavitational bubbles from a 1 mm sonotrode tip resulted in serious acoustic attenuation and high energy in each cavitational bubble being released when the bubbles collapsed, even though the delivered energy density was low [99]. The radiating face of the ultrasonic probe was shown to greatly affect the efficiency of enzyme inactivation in this case. It is of interest that using large sonotrodes may reduce the intensity of bubble collapse and hence are thus not recommended for intensification [99].

The amount of dissolved gas present in media undergoing sonication is a parameter which also influences the acoustic cavitation. Lowering the quantity of dissolved gas reduces the number of nuclei and thereby increases the cavitation threshold and results in a change in the type of cavitation, producing more vaporous cavities [95]. Such microbubbles collapse less violently and hence the rate of sonochemical reaction will be decreased. The selection of the correct ultrasonic frequency for the applications envisaged is also important. Higher frequency or combinations of lower frequencies is recommended for applications controlled by chemical effects whereas lower frequency is recommended for applications with physical effects [73]. The use of multiple transducers can lead to the formation of uniform cavitational behaviour and also minimise the required energy consumption in applied systems. This occurs as it would not be possible to dissipate the same power through a single probe in a large scale bioprocess operation [95]. In addition, the selection of liquids with low vapour pressure, low viscosity and high surface tension was found to enhance cavitational behaviour [95]. Therefore, the rational design of scaled-up ultrasonic reactors requires the quantitative prediction of acoustic streaming, power dissipation, mass transfer and cavitational activity in the reactor by theoretical simulations [19]. An effective scale-up of the ultrasound reactor can be achieved if the energy dissipation pattern in the reactor is known. One of the major disadvantages of ultrasound baths is the directional sensitivity of the ultrasound waves in the bath, which creates a non-homogeneous energy dissipation pattern. This can be overcome by using tubular reactors with ultrasound intensity concentrated at the core of the reactor, but any industrial scale operation has not yet been reported [100]. In an ultrasonic reactor, the input electrical energy undergoes many transformations while being converted into cavitation energy, which is dissipated in the medium to carry out the physical and chemical change. The cavitation intensity varies with the gas content of the medium [101]. The principles described above have influenced the design of several reactor types for ultrasonication-based processes. Coincidence of the compression half cycles of the two acoustic waves while using dual frequency sonochemical reactor has a favourable effect on the production of radicals, as it intensifies the transient collapse of the cavitation bubbles [102]. The dual frequency ultrasonic processor will not be able to contribute sonochemical effects, which is usually attributed to radical formation during transient cavitation. However, the moderate cavitation intensities in dual frequency reactors would favour the physical processes such as extraction and leaching [103]. Cintas et al. [104] used a reverberative flow reactor, in which two irradiation arrays made up of multiple ultrasonic probes were mounted on the upper and lower portion of a metal cuboid reactor, while liquid reactant was allowed to flow through the cuboid space [104, 105].The reflection of ultrasonic waves at the inner wall of the reactor chamber and its reverberation in the chamber caused the acoustic intensity to be multiplied, thereby giving a more non-uniform cavitation field than single probe systems. Similar technology has also been adopted in sonochemical polygonal reactors [106].

A liquid whistle ultrasonic reactor (frequency 5–30 kHz and intensity, 1.5–2.5 W cm^−2^) which causes hydrodynamic cavitation has been widely used for industrial wastewater treatment [107], oil emulsification [108], liquid–solid mixture homogenization [19] and fat hydrolysis [109]. This type of reactor can be operated at low cost and is suitable for continuous flow reactions, with scale-up also possible [19, 104]. With effective reactor design as describe, it is possible to maximise the beneficial effects of ultrasonic intensification in a specific process while minimising cost.

## Kinetic analyses of ultrasonic intensification processes

The modelling of sonoreactors is challenging due to the experimental issues relating to the effects of ultrasound [110]. The kinetic parameters of ultrasonic intensification on chemical degradation, extraction and fermentation such as ultrasonic frequency, ultrasonic intensity, ultrasonic dose and energy output so far reported are given in Table 5. Ultrasonic Intensity (UI) or acoustic energy density (AED) (sound energy per unit volume) can be evaluated calorimetrically via equation 1 as reported by Mason [111] (Table 5). The influence of the probe surface on ultrasonic intensity can also be determined by the equations 2 and 3 (Table 5). Tiehm et al. [116] hypothesised that the ultrasonic intensity related to the power supplied on the transducer area whereas the ultrasonic density related to the sample volume while the ultrasonic dose related to the energy supplied per sample volume. Though the molecular mechanism of sonochemical degradation remains subtle, there is general agreement that bond cleavage arises from the large shear gradient generated throughout the collapse of the cavitation bubbles. During sonochemical processing, once one goes beyond threshold ultrasound intensity, microbubbles are created which increase in size by absorption of acoustic energy until a critical diameter of 250 µm is reached. Such bubbles become unstable and collapse violently within approximately 20 µs. Adiabatic compression raises the internal pressure to about 500 atm while the cavity temperature may reach 5800 K [117]. If a molecular coil was to be placed beneath the imploding cavity it would be dragged along by the microstream towards the interior of the bubble while being strained by the high fluid velocity gradient [114]. However, the hydraulics are significantly different as the interior of the cavity is filled with a compressible gas with a constantly moving boundary and the applied acoustic pressure is not constant but is a sinusoidal function of time [118]. Nguyen et al. [114] have derived an expression to describe the bubble wall motion during implosive collapse as represented by the strain rate distribution ℇ_rr_ using equation 4 (Table 5). Experiments have revealed that the instantaneous radius of the imploding cavity (R) reaches a minimum of the order of 0.5 µm during the final collapse [114]. The strain rate distribution could not be altered other than by changing the ultrasonic conditions. The mechanism of ultrasound assisted acid catalysed transesterification was analysed by the quantitative estimation of physical and chemical effects through simulations of cavitation bubble dynamics. The numerical solution of the bubble dynamics model (see Table 5) can be used to estimate the composition of the bubble contents at the collapse [31]. Ultrasonic intensification can improve the biokinetic parameters such as microbial growth rate [77] during fermentation and also enhance the reaction rate of transesterification during biodiesel production. An investigation of the kinetics of ultrasonic assisted transesterfication of canola waste cooking oil showed that ultrasonication can complete the transesterification within short periods of reaction time and the activation energy was found to be 19, 645 J mol^−1^ K^−1^ [119].

**Table 5:** Kinetic expressions derived for ultrasonic parameters

S.No.	Parameters	Equations	Definitions	References
1	Acoustic energy density (AED)	${\rm AED} = \frac{P}{V}$ $P = mc_{\rm p}{\left(\frac{{\rm d}T}{{\rm d}t}\right)}^{}_{t=0}$	*P* absolute ultrasonic power,* V* volume of the medium (cm^3^ L^−1^),* m* mass,* c*_p_ specific heat capacity, (d*T*/d*t*) range of temperature change during sonication	[111]
2	Ultrasonic intensity (UI) with the influence of diameter of the probe tip	${\rm UI} = \frac{4P}{{D^{2} }}$	*P* absolute ultrasonic power,* D* diameter of the probe tip	[111]
3	Ultrasonic intensity	${\rm UI} = \frac{P}{A}$	UI ultrasonic intensity,* P* ultrasonic power,* A* surface area of the probe	[112]
4	Cell disruption at given acoustic power	$F_{\rm N} = - {\rm exp}\left[ { - \left( {\frac{t}{\alpha }} \right)^{\beta } } \right]$	*F*_N_ cumulative fractions of disrupted cells at given acoustic power, *t* time of ultrasonication,* α* and* β* kinetic constants	[113]
5	Strain rate distribution	$\varepsilon_{{\rm rr}} (r) = - 2 v_{\rm R} R^{2} r^{ - 3}$ $v_{{\rm R}} = {{\rm d}R}/ {{\rm d}t}\, {\dot{Q}}_{\rm a} {\rm O}_2 Z$ $= (2P_{\rm h}/ {3\rho})^{0.5} (R_{\rm m}^3/ R^3-1)^{0.5}$	*ℇ*_rr_ strain rate distribution during cavity collapse,* v*_R_ bubble wall velocity, *ρ* solvent density,* P*_h_ external pressure,* R*_m_ initial radius and* R* instantaneous radius of imploding cavity	[114]
6	Specific Energy input	$E_{\rm s} = \frac{P t}{{V\, {\rm TS}_{0} }}$	*E*_s_ specific energy,* P* ultrasonic power,* t* ultrasonic time,* V* volume of the sample, TS_0_ initial concentration of total solids	[115]
7	Actual energy produced by ultrasonication	$Q_{\rm u} = P \times t$	*Q*_u_ energy output,* P* ultrasonic power,* t* ultrasonic time	[116]
8	Ultrasound dose	${\rm UD}_{0} = \frac{P \times t}{V}$	UD_0_ ultrasonic dose,* P* ultrasonic power,* t* ultrasonic time	[116]
9	Sonochemical effectiveness factor(***e***_us_)	$\varvec{e}_{{{\rm us}}} = f,\eta I,{\mathbb{V}}_{\rm us} ,T$ ${\mathbb{V}}_{\rm us}=V_{\rm us}/V_{\rm tot}$	*f* applied frequency,* ηI* calorimetrically determined power of the transducer,* T* average temperature in the reactor, ${\mathbb{V}}_{\rm us}$ dimensionless cavitationally active volume, *V*_us_ volume of the reactor space affected by sonication,* V*_tot_ total working volume	[110]
10	Bubble dynamics model	Micro-convection: ${\text{V}}_{\text{turb}} ({\text{r}},{\text{t}}) = \frac{{{\text{R}}^{2} }}{{{\text{r}}^{2} }}\left( {\frac{\text{d}R}{\text{d}t}} \right)$	*V*_turb_ velocity of turbulence,* P*_AW_ pressure amplitude of acoustic wave,* R* radius of the bubble, d*R*/d*t* bubble wall velocity,* V*_b_ volume of the bubble,* ρ*_L_ density of the liquid	[31]
		Shock waves: $P_{\rm AW}(r, t)=\rho_{\rm L}\frac{R}{r}\left[ 2\left(\frac{{\rm d}R}{{\rm d}t}\right)^2+R \frac{{\rm d}^{2}R}{{\rm d}t^2}\right]$		

## Conclusion

Although research carried out to date has indicated the utility of ultrasonication for enhancement in numerous applications, there remain many factors that require further analysis for a complete optimisation of ultrasonic-based processes particularly with a focus on improving biofuel yields from either extractive or fermentation processes.

Ultrasonic intensification can offer several advantages during bioprocessing; these include low operating cost [120] compared to other enhancing treatment options, simplicity of operation and modest power requirements. In addition, ultrasonic intensification does not require sophisticated equipment reformatting or intensive technical training for utilisation. Ultrasound would likely improve the productivity of many bioprocesses involving live cells via the enhancement of substrate uptake, enhanced production or growth by increasing cell porosity, and potentially enhanced release of cell components which could have important consequences for volatile components such as bioethanol.

## Abbreviations

AED: acoustic energy density
EPA: eicosapentaenoic acid
UI: ultrasonic Intensity
